# Concurrent preoperative eribulin and radiation for resectable retroperitoneal liposarcoma: a phase 1B study

**DOI:** 10.21203/rs.3.rs-5397300/v1

**Published:** 2024-12-17

**Authors:** Lara Davis, Limin Zhu, Skye C. Mayo, Emile Latour, Byung Park, Wei Huang, Brendan Moloney, Jessica L. Davis, Kristina Wakeman, Brett Sheppard, Kevin G. Billingsley, John Vetto, Cristian D. Valenzuela, Robert L. Eil, Flavio Rocha, Arthur Hung, Christopher W. Ryan

**Affiliations:** Oregon Health & Science University; Oregon Health & Science University; Oregon Health & Science University; Oregon Health & Science University; Oregon Health & Science University; Oregon Health & Science University; Oregon Health & Science University; Indiana University; Oregon Health & Science University; Oregon Health & Science University; Yale Cancer Center; Oregon Health & Science University; Oregon Health & Science University; Oregon Health & Science University; Oregon Health & Science University; Oregon Health & Science University; Oregon Health & Science University

**Keywords:** Sarcoma, Retroperitoneal sarcoma, Radiation therapy, Liposarcoma, Neoadjuvant chemotherapy, Eribulin

## Abstract

**Background:**

Management of retroperitoneal liposarcoma (RPLPS) is challenging and recurrence rates remain high despite aggressive surgical resections. Preoperative radiation alone lacks definitive benefit, thus we sought to evaluate combined chemoradiotherapy with the potential to enhance local efficacy of radiation as well as control micrometastatic disease. We assessed the safety and tolerability of preoperative eribulin, a cytotoxic microtubule inhibitor approved for the treatment of advanced liposarcoma, in combination with radiation in patients with RPLPS.

**Methods:**

In this open-label dose-finding study, patients with primary or recurrent resectable RPLPS received preoperative intensity-modulated radiation therapy (IMRT) with escalating doses of eribulin. Eribulin was administered for three 21-day cycles at a starting dose of 1.1 mg/m^2^. Concurrent radiation to 50.4 Gy began during cycle 1. Surgical resection occurred 3–10 weeks after completion of chemoradiation. The primary endpoint was determination of the recommended phase 2 doses (RP2D) of concurrent eribulin and radiation.

**Results:**

Between 2018–2023, fifteen patients were enrolled. Thirteen patients were evaluable for dose-determination. Four patients treated at starting dose level had no dose-limiting toxicities (DLTs). Two of nine patients treated with escalated eribulin dose had DLTs. The RP2D was established as eribulin 1.4 mg/m^2^ and IMRT 50.4 Gy. Eleven patients were evaluable for secondary efficacy endpoints. The median recurrence-free survival was 30.4 months (95% CI 12.0-NR) and the median overall survival was 54.1 months (95% CI 9.5-NR). Patient reported outcome data did not show any significant changes over the study period.

**Conclusion:**

A preoperative chemoradiation protocol of eribulin in combination with IMRT showed a manageable safety profile and warrants additional prospective evaluation for treatment of resectable RPLPS.

## BACKGROUND

Liposarcoma is the most common soft tissue sarcoma, and the retroperitoneum is one of the most common locations for liposarcoma to arise (1, 2). Over 50% of retroperitoneal sarcomas are liposarcomas (3–6). The complex anatomy of the retroperitoneum and frequent large tumor size make management of retroperitoneal sarcomas particularly challenging, as *en bloc* surgical resection with widely negative margins entails multi-visceral resection and is often difficult to achieve. Despite aggressive surgical resections, rates of locoregional recurrence remain high and repeat resections are frequently required (3, 4). Unlike most other sarcomas, the majority of deaths due to retroperitoneal sarcomas result from uncontrolled or recurrent local disease, and not from distant metastases (7, 8). Thus, there is an urgent need to develop new perioperative treatment strategies for patients with retroperitoneal liposarcoma (RPLPS), since controversy persists regarding the best approach persists.

Several retrospective series suggest a role for preoperative radiation for retroperitoneal sarcomas, and it is standard practice at many large volume sarcoma centers (5, 6, 9, 10). Each center uses varying institutional standards for dose and technique, but most report using IMRT to a dose of 45–50.4 Gy (11). An international, randomized, phase 3 trial comparing preoperative radiotherapy (RT) plus surgery versus surgery alone for patients with primary retroperitoneal sarcoma (EORTC-62092: STRASS) showed similar abdominal recurrence-free survival (ARFS) and overall survival between the two arms as designed (12). However, 28.8% of the patients in the RT plus surgery arm were non-compliant to RT, which may have influenced the study results and subsequent analysis from the trial RT quality-assurance program showed significantly improved ARFS in RT-compliant patients compared to the non-compliant group (13). Further, in a retrospective study of the pooled cohort of patients enrolled in the STRASSS trial as well as off-trial patients who underwent similar treatment, preoperative RT was associated with better AFRS specifically in patients with RPLPS (14). Given the controversy and lack of definitive benefits from preoperative radiation alone, we wished to evaluate a preoperative chemoradiotherapy approach with potential to both enhance local efficacy of radiation and control micrometastatic disease.

Eribulin is FDA-approved for treatment of unresectable or metastatic liposarcoma. Eribulin acts by inhibiting microtubule growth, resulting in G2/M cell cycle arrest and ultimately apoptotic cell death (15). In addition, based on mechanism of action, eribulin is predicted to be a radiosensitizing agent (16). Post-hoc analysis of previous trials in patients with recurrent or metastatic breast cancer have demonstrated that radiation administered during eribulin therapy is safe (17, 18), but the combination has not been studied prospectively before. The aim of this phase 1B clinical trial was to assess the safety and tolerability of preoperative eribulin in combination with radiation in patients with RPLPS.

## METHODS

### Study design and participants

This was an open-label, phase 1B, dose-finding study performed at OHSU Knight Cancer Institute for patients with primary or recurrent resectable retroperitoneal liposarcoma of any subtype. Inclusion criteria included age ≥ 12 years old, Eastern Cooperative Oncology Group (ECOG) performance status 0 or 1, all sites of disease must be resectable or borderline resectable with acceptable morbidity, all sites of disease must be targetable with IMRT with acceptable morbidity, no evidence of distant metastases, no history of prior radiation or chemotherapy for the diagnosis of liposarcoma, and normal organ and marrow function at screening. All patients were reviewed for trial eligibility in our weekly multidisciplinary sarcoma tumor board conference. This trial was approved by the OHSU institutional review board, and was registered at clinicaltrials.gov (NCT03361436).

### Procedures

Escalating doses of eribulin and intensity-modulated radiation therapy (IMRT) were guided by a modified toxicity probability interval (mTPI) design with target toxicity rate of 0.2 (19). Four subjects were enrolled for the first cohort at each dose, and three subjects in subsequent cohorts at the same dose. The dose-limiting toxicity (DLT) observation period for the purpose of dose-determining was Cycles 1–2 (weeks 1– 6). Eribulin was administered on day 1 and 8, every 3 weeks for 3 cycles. Concurrent radiation began during cycle 1, following day 8 eribulin. The starting dose level of eribulin was 1.1 mg/m^2^, with dose level + 1 escalation dose of 1.4 mg/m^2^. The starting dose of IMRT was 50.4 Gy administered over 28 fractions of 180 cGy, with dose level − 1 de-escalation dose of 39.6 Gy if necessitated by DLT. Radiation was administered at OHSU with IMRT in accordance to our protocol which was based on the preliminary consensus guidelines for preoperative radiation for retroperitoneal sarcomas (11). Motion management was done either by Active Breathing Control or 4D CT when the tumor was located near the diaphragm. The Clinical Target Volume was defined as a 1.5 cm expansion around the gross tumor, subtracting any intraabdominal organs or areas outside of the abdominal cavity that did not demonstrate clear invasion. The treatment parameters based off the particular LINAC and motion management techniques employed dictated the Planning Target Volume expansion and was typically an additional 5–10 mm. Areas anticipated to be at high-risk were not given an additional boost sequentially or as an integrated dose. Study schema and dose levels are shown in Fig. 1. Surgical resection occurred within 3–10 weeks after completion of radiation therapy. Subjects will be followed until death or until 10 years after completion of study therapy, whichever occurs first. Full trial protocol available in Supplementary Materials.

### Outcomes

The primary endpoint was to determine the recommended phase 2 doses (RP2D) of concurrent eribulin and radiation. Secondary endpoints included rate of R0 resection, pathologic response, objective response rate (ORR), recurrence free survival (RFS), and overall survival (OS). For exploratory objectives, patients underwent dynamic contrast-enhanced magnetic resonance imaging (DCE-MRI) at three different time points during the preoperative period to evaluate for changes in tumor perfusion/permeability and microenvironment during and after chemoradiation. Two patient-reported outcome questionnaires, the 10 item PROMIS Global Health Scale and 5 item PROMIS Belly Pain Scale, were administered at four time points: baseline, C2D8, prior to surgery and 9 weeks post-op to monitor the overall well-being of study participants (20).

### Statistical analysis

Descriptive statistics were used to describe the demographic and clinical characteristics of the patients (medians and ranges, counts and percentages). DLTs were counted and the RP2D was determined according to interim mTPI monitoring algorithm that was established prior to the study. Adverse events were graded and categorized according to the CTCAE v4.03. All adverse events were tabulated and summarized by major organ category, grade, anticipation, and drug attribution. Median recurrence-free survival with 95% CI were estimated using the Kaplan-Meier method for both local and distant recurrence, as was median overall survival. The proportions of patients that were recurrence-free and the proportions surviving were estimated at 2, 5 and 10 years with 95% CIs. PROMIS symptom measure scores were analyzed across four times points using linear mixed effects models, with a random intercept to account for within-subject correlations. Analysis was performed using R: A Language and Environment for Statistical Computing (21). P < 0.05 was considered to be statistically significant.

Safety was reported on all patients who received at least one dose of eribulin. The dose-determining population consisted of all subjects who remained on protocol therapy through the DLT observation period or discontinued due to DLT. The efficacy population was patients who received all protocol therapy (including 3 cycles of eribulin and radiation) and underwent surgical resection.

## RESULTS

### Patient characteristics and treatment

Between June 2018 and January 2023, fifteen patients with resectable retroperitoneal liposarcoma were enrolled in the study. Demographic and clinical characteristics of the patients are summarized in Table 1. Seven patients were female (46.7%). Patients’ ages were between 44.4 and 79.9 years, with median age of 63.9. Both well-differentiated (40%) and de-differentiated (60%) liposarcoma subtypes were enrolled. Approximately half of all enrolled participants (7 of 15, 46.7%) had a tumor > 15 cm in size at time of enrollment.

All fifteen patients received at least one dose of eribulin and were included in the safety analysis. Two participants stopped protocol therapy prior to completing the DLT observation period due to pandemic-related concerns and thus thirteen patients were evaluable for dose-determination. Eleven patients completed protocol therapy and underwent resection and thus were evaluable for efficacy. The CONSORT diagram of this study is depicted in Fig. 2.

### Safety

The safety profile of preoperative eribulin in combination of IMRT is shown in Table 2. Eleven (73.3%) out of fifteen patients in the safety analysis set had a treatment-related adverse event (TRAE), with eight patients (53.3%) experiencing a grade 3/4 TRAEs. The most frequently reported TRAE of any grade were hematological toxicities, including lymphopenia (53%), leukopenia (40%), anemia (33%), and neutropenia (33%); along with alopecia (53%), radiation dermatitis (40%), and fatigue (33%). The most common high grade TRAEs (≥ grade 3) were lymphopenia (47%), followed by leukopenia (13%), anemia (13%), neutropenia (13%), and hypokalemia (13%). No dose reductions or treatment discontinuation were indicated based on TRAE.

### Recommended Phase 2 Dose Determination

Thirteen patients were evaluable for dose-determining. No DLTs were observed in 4 patients treated at starting dose level (IMRT of 50.4 Gy and eribulin 1.1 mg/m^2^) and thus dosing increased to level 2 (IMRT of 50.4 Gy and eribulin 1.4 mg/m2). Two of nine patients at dose level 2 had DLTs: one febrile neutropenia, and one sudden cardiac death, which was ultimately attributed to a pre-existing cardiac anomaly not related to study treatment. The RP2D was established at eribulin 1.4 mg/m^2^ and IMRT 50.4 Gy over 28 fractions.

### Efficacy

Eleven patients who completed preoperative study treatment and underwent surgery were included for efficacy analysis. Histologic grading of pretreatment biopsy specimens from these 11 patients classified five as well-differentiated, and six as grade 2–3 dedifferentiated. Central pathology review of the resected tumors evaluated resection margin, percentage fibrosis, percentage necrosis, and percent overall treatment as described in Table 3. Nine patients had R0 (81.8%), and two patients had R1 (18.2%) resections (R1 defined as high-grade tumor present at resection margin). Two patients (18.2%) achieved > 30% overall treatment effect.

Patients were followed for a median of 63.5 (95% CI 24.8–65.1) months. The Kaplan Meier estimates of RFS and OS of patients who completed all study treatment are demonstrated in Fig. 3. In total, there were 4 RFS events and 3 OS events. The estimated median RFS was 30.4 months (95% CI 12.0-NR), with 2-year and 5-year RFS of 76.2% (95% CI 33.2% – 93.5%) and 45.7% (95% CI 11.0% – 75.7%) respectively. The estimated median OS was 54.1 months (95% CI 9.5-NR), with 2-year and 5-year OS of 90.0% (95% CI 47.3% – 98.5%) and 38.6% (95% CI 1.4% – 80.9%) respectively.

### Correlative studies

Analysis of PROMIS data at four different time points throughout the study, which include baseline, cycle 2 day 8, prior to surgery, and 9 weeks post-op, did not show any significant changes in global physical health or gastrointestinal pain (Fig. 4). Pharmacokinetic modeling of DCE-MRI data was performed to extract the K^trans^ parameter (MRI contrast agent volume transfer rate constant), a measure of microvascular perfusion and permeability. However, significant motion artifact in most patients limited robust analyses and thus data are not presented.

## DISCUSSION

This is the first prospective study investigating the combination of radiation with eribulin in any disease. The combination of eribulin (1.4 mg/m^2^ on days 1 and 8 every 3 weeks) with radiation (50.4 Gy administered over 28 fractions of 180 cGy) is safe and tolerable in a population of patients with resectable retroperitoneal liposarcoma. Most TRAEs were low grade, with none leading to interruption or discontinuation of study treatment. The overall incidence of TRAEs was comparable to that of the patients who received preoperative radiation only, as reported in the STRASS trial (12).

Although limited by small size, our study showed signals of efficacy, which was one of the secondary trial endpoints. Neoadjuvant radiation with concurrent eribulin resulted in a high proportion of patients having an R0 resection with measurable histologic treatment effect in the majority of patients. Our institution is a high-volume sarcoma center, completing over 50 curative-intent retroperitoneal sarcoma resection surgeries each year, which has been associated with improved disease-free and overall survival (22). Nonetheless, this study reports lower than expected RFS and OS, likely related to small study size and the limited number of events.

When considering preoperative therapy for retroperitoneal sarcomas, the risk of disease progression and worsening of cancer-related symptoms are common concerns among patients and treating physicians. One of fifteen enrolled patients experienced disease progression during preoperative therapy and required early resection. This individual had a large, high-grade inflammatory tumor that liquified while receiving neoadjuvant chemoradiation and underwent resection with R1 margins after cumulative eribulin 5.6 mg/m^2^ (2 cycles) and IMRT 1620 cGy. Patient-reported outcome data collected through PROMIS measures during this study showed that there were no changes in global physical health or gastrointestinal pain before, during, or after the preoperative chemoradiation.

## CONCLUSIONS

Preoperative eribulin with radiation is safe and tolerable for patients with resectable retroperitoneal liposarcoma. Implementation of this treatment approach in a larger prospective trial will be necessary to evaluate the potential improved efficacy of this combination therapy compared to radiation or surgery alone, and whether its benefits extend to all tumor grades.

## Data Availability

The datasets used and/or analyzed during the current study are available from the corresponding author on reasonable request.

## References

[1] Trans-Atlantic RPSWG. Management of primary retroperitoneal sarcoma (RPS) in the adult: a consensus approach from the Trans-Atlantic RPS Working Group. Ann Surg Oncol. 2015;22(1):256–63. 25316486 10.1245/s10434-014-3965-2

[2] Dalal KM, Kattan MW, Antonescu CR, Brennan MF, Singer S. Subtype specific prognostic nomogram for patients with primary liposarcoma of the retroperitoneum, extremity, or trunk. Ann Surg. 2006;244(3):381–91. 16926564 10.1097/01.sla.0000234795.98607.00 PMC1856537

[3] Gronchi A, Miceli R, Shurell E, Eilber FC, Eilber FR, Anaya DA, Outcome prediction in primary resected retroperitoneal soft tissue sarcoma: histology-specific overall survival and disease-free survival nomograms built on major sarcoma center data sets. J Clin Oncol. 2013;31(13):1649–55. 23530096 10.1200/JCO.2012.44.3747

[4] Raut CP, Miceli R, Strauss DC, Swallow CJ, Hohenberger P, van Coevorden F, External validation of a multi-institutional retroperitoneal sarcoma nomogram. Cancer. 2016;122(9):1417–24. 26916507 10.1002/cncr.29931

[5] Kelly KJ, Yoon SS, Kuk D, Qin LX, Dukleska K, Chang KK, Comparison of Perioperative Radiation Therapy and Surgery Versus Surgery Alone in 204 Patients With Primary Retroperitoneal Sarcoma: A Retrospective 2-Institution Study. Ann Surg. 2015;262(1):156–62. 26061213 10.1097/SLA.0000000000001063 PMC4465112

[6] Nussbaum DP, Rushing CN, Lane WO, Cardona DM, Kirsch DG, Peterson BL, Preoperative or postoperative radiotherapy versus surgery alone for retroperitoneal sarcoma: a case-control, propensity score-matched analysis of a nationwide clinical oncology database. Lancet Oncol. 2016;17(7):966–75. 27210906 10.1016/S1470-2045(16)30050-X

[7] Tan MC, Brennan MF, Kuk D, Agaram NP, Antonescu CR, Qin LX, Histology-based Classification Predicts Pattern of Recurrence and Improves Risk Stratification in Primary Retroperitoneal Sarcoma. Ann Surg. 2016;263(3):593–600. 25915910 10.1097/SLA.0000000000001149 PMC4619189

[8] Gronchi A, Strauss DC, Miceli R, Bonvalot S, Swallow CJ, Hohenberger P, Variability in Patterns of Recurrence After Resection of Primary Retroperitoneal Sarcoma (RPS): A Report on 1007 Patients From the Multi-institutional Collaborative RPS Working Group. Ann Surg. 2016;263(5):1002–9. 26727100 10.1097/SLA.0000000000001447

[9] Lane WO, Cramer CK, Nussbaum DP, Speicher PJ, Gulack BC, Czito BG, Analysis of perioperative radiation therapy in the surgical treatment of primary and recurrent retroperitoneal sarcoma. J Surg Oncol. 2015;112(4):352–8. 26238282 10.1002/jso.23996

[10] Ruff SM, Heh V, Konieczkowski DJ, Onuma A, Dunlop HM, Kim AC, Radiation therapy for retroperitoneal sarcoma: practice patterns in North America. Radiat Oncol. 2024;19(1):38. 38491404 10.1186/s13014-024-02407-8 PMC10943830

[11] Baldini EH, Wang D, Haas RL, Catton CN, Indelicato DJ, Kirsch DG, Treatment Guidelines for Preoperative Radiation Therapy for Retroperitoneal Sarcoma: Preliminary Consensus of an International Expert Panel. Int J Radiat Oncol Biol Phys. 2015;92(3):602–12. 26068493 10.1016/j.ijrobp.2015.02.013

[12] Bonvalot S, Gronchi A, Le Pechoux C, Swallow CJ, Strauss D, Meeus P, Preoperative radiotherapy plus surgery versus surgery alone for patients with primary retroperitoneal sarcoma (EORTC-62092: STRASS): a multicentre, open-label, randomised, phase 3 trial. Lancet Oncol. 2020;21(10):1366–77. 32941794 10.1016/S1470-2045(20)30446-0

[13] Haas R, Stelmes JJ, Zaffaroni F, Sauve N, Clementel E, Bar-Deroma R, Critical impact of radiotherapy protocol compliance and quality in the treatment of retroperitoneal sarcomas: Results from the EORTC 62092 – 22092 STRASS trial. Cancer. 2022;128(14):2796–805. 35536104 10.1002/cncr.34239

[14] Callegaro D, Raut CP, Ajayi T, Strauss D, Bonvalot S, Ng D, Preoperative Radiotherapy in Patients With Primary Retroperitoneal Sarcoma: EORTC-62092 Trial (STRASS) Versus Off-trial (STREXIT) Results. Ann Surg. 2023;278(1):127–34. 35833413 10.1097/SLA.0000000000005492

[15] Smith JA, Wilson L, Azarenko O, Zhu X, Lewis BM, Littlefield BA, Eribulin binds at microtubule ends to a single site on tubulin to suppress dynamic instability. Biochemistry. 2010;49(6):1331–7. 20030375 10.1021/bi901810u PMC2846717

[16] Benlloch R, Castejon R, Rosado S, Coronado MJ, Sanchez P, Romero J. In vitro radiosensitization by eribulin in human cancer cell lines. Rep Pract Oncol Radiother. 2022;27(3):509–18. 36186704 10.5603/RPOR.a2022.0049 PMC9518772

[17] Yardley D, Vahdat L, Rege J, Cortés J, Wanders J, Twelves C. Abstract P6-11-14: Post-hoc safety and tolerability assessment in patients receiving palliative radiation during treatment with eribulin mesylate for metastatic breast cancer. Cancer Research. 2012;72(24_Supplement):P6-11-4-P6-4.

[18] Meattini I, Desideri I, Di Cataldo V, Francolini G, De Luca Cardillo C, Scotti V, Safety of eribulin mesylate and concomitant radiotherapy for metastatic breast cancer: a single-center experience. Future Oncol. 2016;12(9):1117–24. 26956105 10.2217/fon-2015-0059

[19] Ji Y, Wang SJ. Modified toxicity probability interval design: a safer and more reliable method than the 3 + 3 design for practical phase I trials. J Clin Oncol. 2013;31(14):1785–91. 23569307 10.1200/JCO.2012.45.7903 PMC3641699

[20] Cella D, Riley W, Stone A, Rothrock N, Reeve B, Yount S, The Patient-Reported Outcomes Measurement Information System (PROMIS) developed and tested its first wave of adult self-reported health outcome item banks: 2005–2008. J Clin Epidemiol. 2010;63(11):1179–94. 20685078 10.1016/j.jclinepi.2010.04.011 PMC2965562

[21] Team RC. R: A Language and Environment for Statistical Computing. Vienna, Austria. R Foundation for Statistical Computing; 2024.

[22] Callegaro D, Barretta F, Raut CP, Johnston W, Strauss DC, Honore C, New Sarculator Prognostic Nomograms for Patients With Primary Retroperitoneal Sarcoma: Case Volume Does Matter. Ann Surg. 2024;279(5):857–65. 37753660 10.1097/SLA.0000000000006098

